# Lessons from 20 years of curative therapy of childhood acute leukaemia.

**DOI:** 10.1038/bjc.1992.32

**Published:** 1992-02

**Authors:** D. Pinkel

**Affiliations:** Kana Research Chair in Pediatric Leukemia, University of Texas M.D. Anderson Cancer Center, Houston 77030.

## Abstract

The past 20 years of curative therapeutics of childhood acute leukaemia has been largely a period of consolidation of gains, refinement of techniques and dissemination of expertise and technology. However, certain lessons have been learned. First, cure can be permanent but the complexity and cost of curative treatment currently restricts its accessibility; prevention or simple curative treatment is needed. Secondly, cure of the child demands that the risk of adverse sequelae of treatments be carefully balanced with known therapeutic benefits. Thirdly, preventive meningeal irradiation is no longer required. Fourth, treatment intensification is self-limiting. Adverse reactions can cancel out or exceed therapeutic benefits, resulting in a lower cure rate or a similar cure rate with lower quality of cure. Finally, morphology, immunophenotype and genotype of acute leukaemia are important criteria for selecting and scheduling drug therapy. Genotype may be the most important since leukaemia is a genetic disorder for which morphology and immunophenotype are mere reflections. However, none of these features, individually or together, are sufficient to explain all the difference in outcome among children on a given treatment plan or to completely fulfill the need of criteria for selection of treatment. Acute leukaemia remains an unsolved problem demanding considerably more basic and clinical research to meet the need for prevention and simple dependable curative treatment.


Br. J. Cancer (1992), 65, 148–153 © Macmillan Press Ltd., 1992

REVIEW

### Lessons from 20 years of curative therapy of childhood acute leukaemia*

D. Pinkel

*Kana Research Chair in Pediatric Leukemia, The University of Texas M.D. Anderson Cancer Center, 1515 Holcombe Boulevard, Houston, Texas 77030, USA.*

Twenty years have passed since the first reports that childhood acute lymphoid leukaemia was no longer incurable (Aur, *et al.*, 1971; Pinkel, 1971*a*). A combination chemotherapy and irradiation program consisting of 1 month of remission induction, 1 week of intensive intravenous chemotherapy, 3 weeks of preventive meningeal therapy and 2 to 3 years of continuation chemotherapy resulted in lengthy leukaemia-free survival in one half of the patients. Lancet (1972) called it 'radical treatment,' but approvingly so. The purpose of this essay is to describe some of the important lessons about curing acute leukaemia that have been learned during the past two decades. The lessons discussed will concern the permanence and prevalence of cure, the cure of the child as well as of the leukaemia, the discarding of preventive meningeal irradiation, the limits of intensification of treatment, and the importance of morphology, immunophenotype and genotype in selection and scheduling of treatment.

### Permanence and prevalence of cure

The potential permanence of cure of acute lymphoid leukaemia (ALL) was established when it was demonstrated that relapse was rare in children who experienced continuous complete remission for 7 years and were off therapy for 4 years (Pinkel, 1979). Although the frequency of cure is less in acute myeloid leukaemia (AML), durable complete remission is also evident under similar circumstances (Mirro *et al.*, 1990). Figure 1 illustrates the event-free survival of the children with ALL in a 1967–68 study, the first to yield a 50% cure rate (Aur *et al.*, 1971; Pinkel, 1987). These results have been reproduced in numerous institutional and cooperative group studies and there is general consensus that permanent cures can result from modern treatment.

The more difficult issue is the prevalence of cure. National biostatistics in the United States and the United Kingdom indicate that childhood mortality from acute leukaemia has been halved in the past 20 years while incidence is unchanged (Miller & McKay, 1984; Birch *et al.*, 1988). Therefore, curative therapy can be assumed to be delivered to most children with leukaemia. However, this does not appear to be the situation in developing nations where child health care is severely limited or services are rationed according to cost/benefit ratio. Although accurate statistics are unavailable, it is likely that less than 10% are cured and they are likely the children of the more privileged. Thus, the challenge remains to prevent childhood leukaemia or develop a simple cure if worldwide childhood leukaemia mortality is to be significantly affected by modern science.

### Cure of the child as well as of the leukaemia

From the earliest attempts to cure childhood leukaemia paediatricians have been concerned about cure of the child as well as of the leukaemia. Anthropometric and neuropsychological parameters, school performance and social and work adjustment have been assessed during and after cessation of treatment. The children have been closely observed for second cancers and organ failures that might be attributed to the leukaemia or its treatment. Efforts have been made to establish relationships between adverse sequelae of leukaemia and specific treatment agents in order to evaluate the relative human cost/benefit ratios of these agents. For the most part, children treated with conventional antimetabolite therapy demonstrate relatively normal growth and development, a low risk of delayed sequelae and good adjustment to maturation.

Current information indicates that the most hazardous agents in children with leukaemia are radiation therapy, alkylating drugs such as cyclophosphamide, anthracyclines such as doxorubicin, and epipodophyllotoxins such as etoposide. Radiation therapy produces growth inhibition and secondary neoplasms. Cyclophosphamide causes sterility, bladder fibrosis and carcinomas. In one report more than one-half of children surviving ALL who had received doxorubicin demonstrated clinically significant cardiomyopathy 5 to 15 years later (Lipshultz *et al.*, 1991). Etoposide and teniposide, used in high total cumulative doses, have been implicated in secondary acute leukaemia, often demonstrating chromosomal translocations with an 11q23 breakpoint (Ratain *et al.*, 1987).

The current therapeutic intervention in childhood leukaemia exhibiting the most severe adverse sequelae is total body irradiation (TBI), myeloablative chemotherapy and allogeneic bone marrow transplantation (Kolb & Bender-

**Figure 1** Event-free survival, St Jude Study V. Of 35 children with acute lymphoid leukaemia admitted to this study in 1967–68, 18 survive in initial complete remission 20 years after cessation of treatment (Pinkel, 1989).

*From a lecture presented at the celebration of the new paediatric oncology unit at St. Bartholomew's Hospital, London, 13 July 1990.
Received 3 June 1991; and in revised form 7 October 1991.

Gotze, 1990). All the survivors experience growth failure, 40% develop chronic graft *vs* host disease and 70% have gonadal failure. Other frequent sequelae are renal, hepatic and pulmonary insufficiency and multiple endocrine disorders. The inherent problem with this modality is that all organs and tissues are severely injured by the pre-transplant conditioning but only one, the hematopoietic bone marrow, is replaced. Except for the graft *vs* host disease, the TBI and myeloablative chemotherapy preceding marrow autografts result in similar sequelae.

In summary, during the past 20 years much has been learned about the adverse sequelae of leukaemia therapy. These can result in children cured of leukaemia, but possibly not cured as children. They can be burdened permanently with a legacy of growth, endocrine and neuropsychological disturbances, organ failure and high risk for second cancers. For this reason it is obligatory that the risks of specific agents or modalities precluding cure of the child be weighed carefully with their potential benefit in prolonging leukaemia-free survival. When treatment with high risk of serious adverse sequelae is little or no more effective than low risk treatment, the low risk treatment needs to be chosen because it is the more curative.

**Figure 2** Groups of DBA2 mice bearing L1210 leukaemia were given various dosages of methotrexate (amethopterin) and their survivals measured. Increased dosage resulted in increased survival until excessive dosage and its toxicity led to shortened survival (Goldin *et al.*, 1956).

### Discarding preventive meningeal irradiation

One of the key features of successful curative therapy 20 years ago was meningeal irradiation, with or without intrathecal chemotherapy, to prevent primary meningeal relapse (Aur *et al.*, 1971). Its value was demonstrated in a randomised comparative study initiated shortly after a pilot study suggested it. However, a subsequent comparative study by the Paediatric Oncology Group (POG) revealed that a three drug combination of methotrexate, hydrocortisone, and cytarabine, injected intrathecally during remission induction and periodically during the next 2 years of continuation chemotherapy, was therapeutically equivalent to cranial irradiation and intrathecal methotrexate (Sullivan, *et al.*, 1982). In a recent POG pilot study in which intermediate high dose methotrexate and cytarabine were administered as well as three drug intrathecal therapy, only three of 99 patients developed primary meningeal relapse (Krance *et al.*, 1991). In the last 5 years of experience at M.D. Anderson Cancer Center there has been no instance of isolated primary meningeal relapse in children with acute leukaemia who received intrathecal therapy and intermediate high dosage intravenous chemotherapy.

In conclusion, meningeal irradiation with its consequent risks of neuropsychological, growth and neoplastic sequelae, can be omitted when appropriate systemic and intrathecal chemotherapy are utilised.

### The limits of intensification of treatment

An important lesson learned in the past 20 years is the self-limiting therapeutic value of increasing the number and dosage of anti-leukaemia agents. Goldin and his colleagues first demonstrated the practical limits of treatment intensification in leukaemia (Figure 2) (Goldin *et al.*, 1956). Their experiments, using a transplantable leukaemia in inbred mice, showed that increasing the dosage of methotrexate improved survival up to a certain point, beyond which there was progressive shortening of survival with serial increases. There was an optimal dosage of drug above which therapeutic effect was surpassed by toxic effect.

A late 1960's study in children with ALL demonstrated that full, more toxic dosage combination chemotherapy resulted in longer remissions than half, less toxic dosage (Pinkel *et al.*, 1971*b*). The need for 'maximum tolerable dosage' was emphasised, therefore, in the curative treatment programs that followed. However, in a 1972–75 study, children with ALL who received two drugs at maximum tolerable dosage had a better chance of surviving in complete remission than those receiving three or four drugs (Figure 3) (Aur *et al.*, 1978). Although the numerical difference was insufficient to be statistically significant, the results suggested that intensification of chemotherapy by use of more drugs might be nonproductive, especially if the additional drugs were less effective and their toxicity overlapped with that of the more effective agents, thus inhibiting their dosage.

Non-comparative studies of ALL have been described in which better remission experience is claimed for use of multiple drugs, including anthracyclines and alkylating agents, rather than simpler antimetabolite regimens (reviewed by Rivera & Mauer, 1987). However, the claims are based largely on historical comparisons, it is not clear whether the patients on the simpler regimens received maximum tolerable dosage, and the impact of self-exclusion of patients who are sicker, poorer or less nourished from the multiple drug regimens is not quantitated. On the other hand, the POG 8205, 8399 and 8602 studies focus on optimal dosage of antimetabolites (methotrexate, mercaptopurine, cytarabine),

**Figure 3** Children with acute lymphoid leukaemia were randomised to one of four treatment regimens after remission induction and preventive meningeal therapy. Those receiving methotrexate and mercaptopurine experienced better leukaemia-free survival than those receiving additional drugs (Aur *et al.*, 1978).

largely administered parenterally and in intermediate high as well as conventional dosage; they exclude anthracyclines, alkylating agents and radiation (Camitta *et al.*, 1989; Land *et al.*, 1989; Krance *et al.*, 1991). These studies have yielded therapeutic results historically comparable to those of the more extensive multiple drug regimens, but without the liability of their potential adverse sequelae.

In summary, there is currently no information to indicate that maximum tolerable dosage of antimetabolite therapy, as currently administered, is of less efficacy in ALL than more extensive multiple drug regimens. However, as indicated further on in this review, this may only be true for the predominating B-precursor ALL of childhood.

In the case of acute nonlymphoid leukaemia, the limits of treatment intensification are more apparent. In three consecutive treatment protocols at one leukaemia center the dosage and number of drugs and the consequent toxicity were markedly increased (Figure 4a) (Mirro *et al.*, 1990). However, the plateaus of continuous complete remission for children who received relatively simple, nontoxic, outpatient treatment in earlier years are not significantly different from those who were given complex, highly toxic inpatient-based treatment more recently. The results of consecutively more intense POG treatment programs are strikingly similar (Figure 4b) (Steuber *et al.*, 1990). Although higher remission induction rates improved short term survival, long term leukaemia-free survival remains approximately the same.

The ultimate in intensification of leukaemia therapy is myeloablative chemotherapy, usually accompanied by lethal TBI, followed by allogeneic, mismatched, or autologous bone marrow transplantation. Again, there appears to be no therapeutic advantage of this method over present current combination chemotherapy regimens that bear considerably less cost in adverse sequelae (Pinkel, 1989*a*). This has been demonstrated for ANLL in first remission in children and young adults (Figure 5) (Dahl *et al.*, 1990; Geller *et al.*, 1990; Schiller *et al.*, 1990) and for ALL in first remission in children and young adults (Figure 6) (Chessels *et al.*, 1990; Horowitz *et al.*, 1991). In addition, a recent study of the Children's Cancer Study Group is reported to show no significant difference in 2 year event-free survival for a large group of children with ANLL in first remission who received myeloablation and allogeneic marrow transplant *vs* chemotherapy alone (Lampkin *et al.*, 1990). When ALL in second remission is treated with myeloablation and autografts of preserved 'purged' marrow event-free survival is almost identical to that with chemotherapy alone, when compared historically (Figure 7) (Rivera *et al.*, 1986; Sallan *et al.*, 1989).

Taken together, the data suggest that once maximum tolerated dosage of appropriate chemotherapy is achieved further intensification in amount or variety of agents produces more immediate toxicity and late adverse sequelae without improving curability. As in Goldin's mice, treatment intensification becomes self-limiting in its therapeutic value.

**Figure 5a** The lengthy complete remission experience of children with acute myeloid leukaemia in first remission was not significantly different for allogeneic marrow transplants *vs* chemotherapy alone (Dahl *et al.*, 1990). **b**, Young adults in first remission of acute myeloid leukaemia had similar cure rates whether treated with chemotherapy-conditioned allogeneic marrow transplants or intensive chemotherapy alone (Geller *et al.*, 1990).

**Figure 4a** Increasing number and dosage of drugs were used in treatment programs for children with acute myeloid leukaemia at St Jude Children's Research Hospital from 1976 to 1986. The consequence was greater toxicity and more hospitalisation without improvement in cure rate (Mirro *et al.*, 1990). **b**, Consecutive treatment regimens of the Paediatric Oncology Group for children with acute myeloid leukaemia show similar cure rates despite improvements in short term survival (Steuber *et al.*, 1990).

### The importance of morphology, immunophenotype and genotype in selection and scheduling of treatment

Early in the development of chemotherapy of acute leukaemia it became apparent that acute myeloid leukaemia (AML) was less responsive to prednisone, methotrexate and mercaptopurine than ALL. When daunorubicin and cytosine arabinoside became available and were found to be highly effective for inducing remission of AML, it became apparent that morphology was a key determinant in selection of anti-leukaemia drugs. In the 1970's it became standard practice to use different treatment protocols for AML *vs* ALL. Among the morphological subtypes of AML, the monocytoid and myelomonocytoid varieties appeared to be more sensitive to the epipodophyllotoxins, etoposide and teniposide.

In 1975 thymic precursor ALL was identified as a distinct clinical and biological subclass of morphological ALL, associated with short remissions and a low cure rate when treated

**Figure 6** Leukaemia-free survival (LFS) in young German adults with acute lymphoid leukaemia was similar whether treated with chemotherapy alone or myeloablation and allogeneic marrow transplant (Horowitz *et al.*, 1991).

**Figure 7a** Second remissions of acute lymphoid leukaemia in children treated with chemotherapy depended on duration of first remission (Rivera *et al.*, 1986). —— first remission ⩾ 18 months; --- first remission < 18 months). **b**, Remission experience of comparable children who received myeloablation and purged marrow autografts was no different (Sallan *et al.*, 1989).

according to standard protocols for ALL (Sen & Borella, 1975). The hypothesis was proposed that immunophenotype might be a determinant of chemotherapy sensitivity of ALL and a criterion for selection of drug treatment (Pinkel, 1979). This hypothesis was supported by studies in mice which demonstrated that thymic leukaemia was more sensitive to cyclophosphamide and cytarabine than methotrexate and mercaptopurine (Frei *et al.*, 1974). A comparative clinical trial demonstrated that children with thymic ALL who received cyclophosphamide and cytarabine in addition to methotrexate and mercaptopurine had a superior cure rate while those with non-thymic ALL did not (Figure 8) (Lauer *et al.*, 1987). Reports concerning B cell lymphoma and B cell ALL have emphasised the prime importance of cyclophosphamide in this disorder. Currently, the POG and others utilise separate treatment protocols for T cell, B cell and B precursor ALL, verifying the significance of immunophenotype in selection and scheduling of ALL therapy.

The importance of genotype in selecting treatment was recognised in the same way as the significance of morphology and immunophenotype had been identified earlier. Children with pseudodiploid ALL, with chromosomal translocations, particularly t(1;19), t(4;11) and t(9;22), in their leukaemia cell metaphases, were noted to have shorter remissions and a lower cure rate (Pui *et al.*, 1988). On the other hand, those with hyperdiploid ALL, with chromosome numbers above 50 and/or a DNA index greater than 1.16 in their leukaemia cells, had superior remission durations and cure rates on the same treatment regimens. These observations and others led to the hypothesis that antileukaemia drugs should be selected according to genotype (Pinkel, 1987). Supporting this hypothesis are the following: acute leukaemias are genetic disorders of hematopoiesis; their morphology and immunophenotype are reflections of these genetic disorders; the curative antileukaemia agents generally act through modification of DNA synthesis and structure; cure of acute leukaemia results in eradication of the genetically disordered leukaemia cells but not the genetically normal hematopoietic

**Figure 8a** Children with non-T lymphoid leukaemia were not benefited by addition of cyclophosphamide and cytarabine to their continuation chemotherapy (Lauer *et al.*, 1987). **b**, For T-cell lymphoid leukaemia, only children who received cyclophosphamide and cytarabine remained in remission (Lauer *et al.*, 1987).

cells of similar morphology and immunophenotype. Further support for this concept is the observation that acute promyeloid leukaemia with the 15;17 translocation and aberrant messenger RNA for retinoic acid receptor alpha is uniquely responsive to all trans retinoic acid, albeit through a differentiation mechanism (Huang *et al.*, 1988; de Thé *et al.*, 1990). In pilot studies described elsewhere the idea that genotype may be the most important criterion of selecting and scheduling antileukaemia drugs is being explored (Pinkel, 1989*b*). A new POG phase 3 comparative study of B precursor ALL uses hyperdiploidy, as indicated by DNA index, to stratify patients.

However, none of these features – morphology, immunophenotype or genotype – completely explain differences in curability with specific treatment regimens. Some children with hyperdiploid B precursor ALL experience relapse after treatment that is curative for the vast majority with similar disease. On the other hand, some children with ALL who have translocations associated with poor outcome are cured with treatment that is unsuccessful in most children with the same findings. Obviously, there are important factors in selecting therapy that are yet to be determined.

### Summary

The past 20 years of curative therapeutics of childhood acute leukaemia has been largely a period of consolidation of gains, refinement of techniques and dissemination of expertise and technology. However, certain lessons have been learned. First, cure can be permanent but the complexity and cost of curative treatment currently restricts its accessibility; prevention or simple curative treatment is needed.

Secondly, cure of the child demands that the risk of adverse sequelae of treatments be carefully balanced with known therapeutic benefits. Thirdly, preventive meningeal irradiation is no longer required. Fourth, treatment intensification is self-limiting. Adverse reactions can cancel out or exceed therapeutic benefits, resulting in a lower cure rate or a similar cure rate with lower quality of cure. Finally, morphology, immunophenotype and genotype of acute leukaemia are important criteria for selecting and scheduling drug therapy. Genotype may be the most important since leukaemia is a genetic disorder for which morphology and immunophenotype are mere reflections. However, none of these features, individually or together, are sufficient to explain all the difference in outcome among children on a given treatment plan or to completely fulfill the need of criteria for selection of treatment.

Acute leukaemia remains an unsolved problem demanding considerably more basic and clinical research to meet the need for prevention and simple dependable curative treatment.

### References

AUR, R.J.A., SIMONE, J.V., VERZOSA, M.S. & 8 others (1978). Childhood acute lymphocytic leukemia, Study VIII. *Cancer*, **42**, 2123.

AUR, R.J.A., SIMONE, J., HUSTU, H.O. & 4 others (1971). Central nervous system therapy and combination chemotherapy of childhood lymphocytic leukemia. *Blood*, **37**, 272.

BIRCH, J.M., MARSDEN, H.B., JONES, P.H., MORRIS-JONES, P.H., PEARSON, D. & BLAIR, V. (1988). Improvements in survival from childhood cancer: results of a population based survey over 30 years. *Br. Med. J.*, **296**, 1372.

CAMITTA, B., LEVENTHAL, B., LAUER, S. & 9 others (1989). Intermediate-dose intravenous methotrexate and mercaptopurine therapy for non-T, non-B acute lymphocytic leukemia of childhood: a Pediatric Oncology Group study. *J. Clin. Oncol.*, **7**, 1539.

CHESSELLS, J.M., BAILEY, C.C. & RICHARDS, S. (1990). Bone marrow transplantation (BMT) in first remission for children with high risk lymphoblastic leukemia (ALL): the UK experience. *Blood*, **??**, 533a.

DAHL, G.V., KALWINSKY, D.K., MIRRO, JR. J. & 8 others (1990). Allogeneic bone marrow transplantation in a program of intensive sequential chemotherapy for children and young adults with acute nonlymphocytic leukemia in first remission. *J. Clin. Oncol.*, **8**, 295.

DE THÉ, H., CHOMIENNE, C., LANOTTE, M., DEGOS, L. & DEJEAN, A. (1990). The t(15;17) translocation of acute promyelocytic leukaemia fuses the retinoic acid receptor α gene to a novel transcribed locus. *Nature*, **347**, 558.

EDITORIAL (1972). Radical treatment of acute leukaemia in childhood. *Lancet*, **ii**, 910.

FREI, III, E., SCHABEL, JR., F.M. & GOLDIN, A. (1974). Comparative chemotherapy of AKR lymphoma and human hematological neoplasia. *Cancer Res.*, **34**, 184.

GELLER, R.B., SARAL, R., KARP., J.E., SANTOS, G.W. & BURKE, P.J. (1990). Cure of acute myelocytic leukemia in adults: a reality. *Leukemia*, **4**, 313.

GOLDIN, A., VENDITTI, J.M., HUMPHREYS, S.R. & MANTEL, N. (1956). Modification of treatment schedules in the management of advanced mouse leukemia with amethopterin. *J. Natl Cancer Inst.*, **17**, 203.

HOROWITZ, M.M., MESSERER, D., HOELZER, D. & 20 others (1991). Chemotherapy compared with bone marrow transplantation for adults with acute lymphoblastic leukemia in first remission. *Ann. Intern. Med.*, **115**, 13.

HUANG, M.E., YU-CHEN, Y., SHU-RONG, C. & 4 others (1988). Use of all-trans retinoic acid in the treatment of acute promyelocytic leukemia. *Blood*, **72**, 567.

KOLB., H.J. & BENDER-GOTZE, C. (1990). Late complications after allogeneic bone marrow transplantation for leukaemia. *Bone Marrow Transplant.*, **6**, 61.

KRANCE, R.A., NEWMAN, E.M., RAVINDRANATH & 8 others (1991). A pilot study of intermediate dose methotrexate and ara-c 'spread-out' or 'up-front' in continuation therapy for childhood non-T, non-B acute lymphoblastic leukemia. *Cancer*, **67**, 550.

LAMPKIN, B., WELLS, R., WOODS, W. & 16 others (1990). Preliminary results: transplantation (BMT) *vs* intensification chemotherapy (Itf) and maintenance chemotherapy (M) *vs* no M in childhood acute nonlymphocytic leukemia (ANL). *Proc ASCO*, **9**, 216.

LAND, V.J., PULLEN, D.J., SHUSTER, J.J., ALVARADO, C., AMYLON, M. & HARRIS, M.B. (1989). Continuing improvement of outcome in childhood non-T, non-B acute lymphocytic leukemia (NTNB-ALL): Pediatric Oncology Group (POG) experience in the 1980's. *Blood*, **74**, 80a.

LAUER, S.J., PINKEL, D., BUCHANAN, G. & 8 others (1987). Cytosine arabinoside/cyclophosphamide pulses during continuing therapy for childhood acute lymphoblastic leukemia. *Cancer*, **60**, 2366.

LIPSHULTZ, S.E., COLAN, S.D., GELBER, R.D., PEREZ-ATAYDE, A.R., SALLAN, S.E. & SANDERS, S.P. (1991). Late cardiac effects of doxorubicin therapy for acute lymphoblastic leukemia in childhood. *N. Engl. J. Med.*, **324**, 808.

MILLER, R.W. & MCKAY, F.W. (1984). Decline in US childhood cancer mortality 1950 through 1980. *JAMA*, **251**, 1567.

MIRRO, J., CROM, W., SANTANA, V.M. & 4 others (1990). AML trials at St. Jude Children's Research Hospital. *Acute Myelogenous Leukemia: Progress and Controversies*. Gale, R.P., (ed.) p. 219. Wiley-Liss, Inc.

PINKEL, D. (1971*a*). Five year follow-up of 'total therapy' of childhood lymphocytic leukemia. *JAMA*, **216**, 648.

PINKEL, D., HERNANDEZ, K., BORELLA, L. & 4 others (1971*b*). Drug dosage and remission duration in childhood lymphocytic leukemia. *Cancer*, **27**, 247.

PINKEL, D. (1979). The Ninth Annual David Karnofsky Lecture: Treatment of acute lymphocytic leukemia. *Cancer*, **43**, 1128.

PINKEL, D. (1987). Curing children of leukemia. *Cancer*, **59**, 1683.

PINKEL, D. (1989*a*). Allogeneic bone marrow transplantation in children with acute leukemia: a practice whose time has gone. *Leukemia*, **3**, 242.

PINKEL, D. (1989*b*). Species-specific therapy of acute lymphoid leukemia. *Modern Trends in Human Leukemia VIII*. Neth, R. *et al.* (eds). p. 27. Springer-Verlag: NY.

PUI, C.-H., WILLIAMS, D.L., ROBERSON, P.K. & 8 others (1988). Correlation of karyotype and immunophenotype in childhood acute lymphoblastic leukemia. *J. Clin. Oncol.*, **6**, 56.

RATAIN, M.J., KAMINER, L.S. & BITRAN, J.D. (1987). Acute nonlymphocytic leukemia following etoposide and cisplatin combination chemotherapy for advanced non-small-cell carcinoma of the lung. *Blood*, **70**, 1412.

RIVERA, G.K. & MAUER, A.M. (1987). Controversies in the management of childhood acute lymphoblastic leukemia: treatment intensification, CNS leukemia, and prognostic factors. *Semin. Hematol.*, **24**, 12.

RIVERA, G.K., BUCHANAN, G., BOYETT, J.M. & 6 others (1986). Intensive retreatment of childhood acute lymphoblastic leukemia in first bone marrow relapse. *N. Engl. J. Med.*, **315**, 273.

SALLAN, S.E., NIEMEYER, C.M., BILLETT, A.L. & 8 others (1989). Autologous bone marrow transplantation for acute lymphoblastic leukemia. *J. Clin. Oncol.*, **7**, 1594.

SCHILLER, G.J., NIMER, S.D., TERRITO, M.C., HO, W.G. & CHAMPLIN, R.E. (1990). A controlled study comparing bone marrow transplant versus high-dose cytarabine-based consolidation chemotherapy for acute myelogenous leukemia in first remission. *Blood*, **76**, 563a.

SEN, L. & BORELLA, L. (1975). Clinical importance of lymphoblasts with T markers in childhood acute leukemia. *N. Engl. J. Med.*, **292**, 828.

STEUBER, C.P., KRISCHER, J., CULBERT, S. & 4 others (1990). Prognostic factors and treatment outcome in childhood acute myeloid leukemia (AML): the POG experience. In *Acute Myelogenous Leukemia: Progress and Controversies*. Gale, R.P. (ed). p. 193. Wiley-Liss, Inc. NY.

SULLIVAN, M.P., CHEN, T., DYMENT, P.G., HVIZDALA, E. & STEUBER, C.P. (1982). Equivalence of intrathecal chemotherapy and radiotherapy as central nervous system prophylaxis in children with acute lymphatic leukemia: a Pediatric Oncology Group study. *Blood*, **60**, 948.


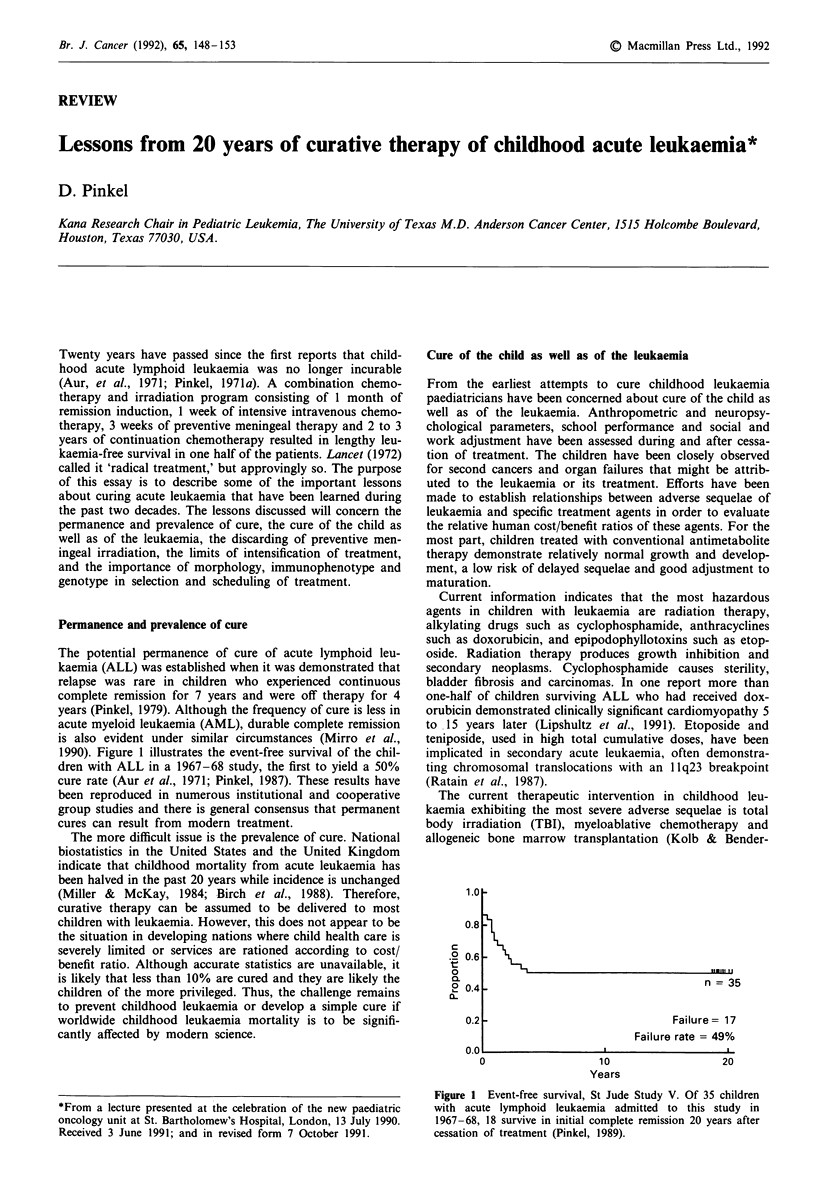

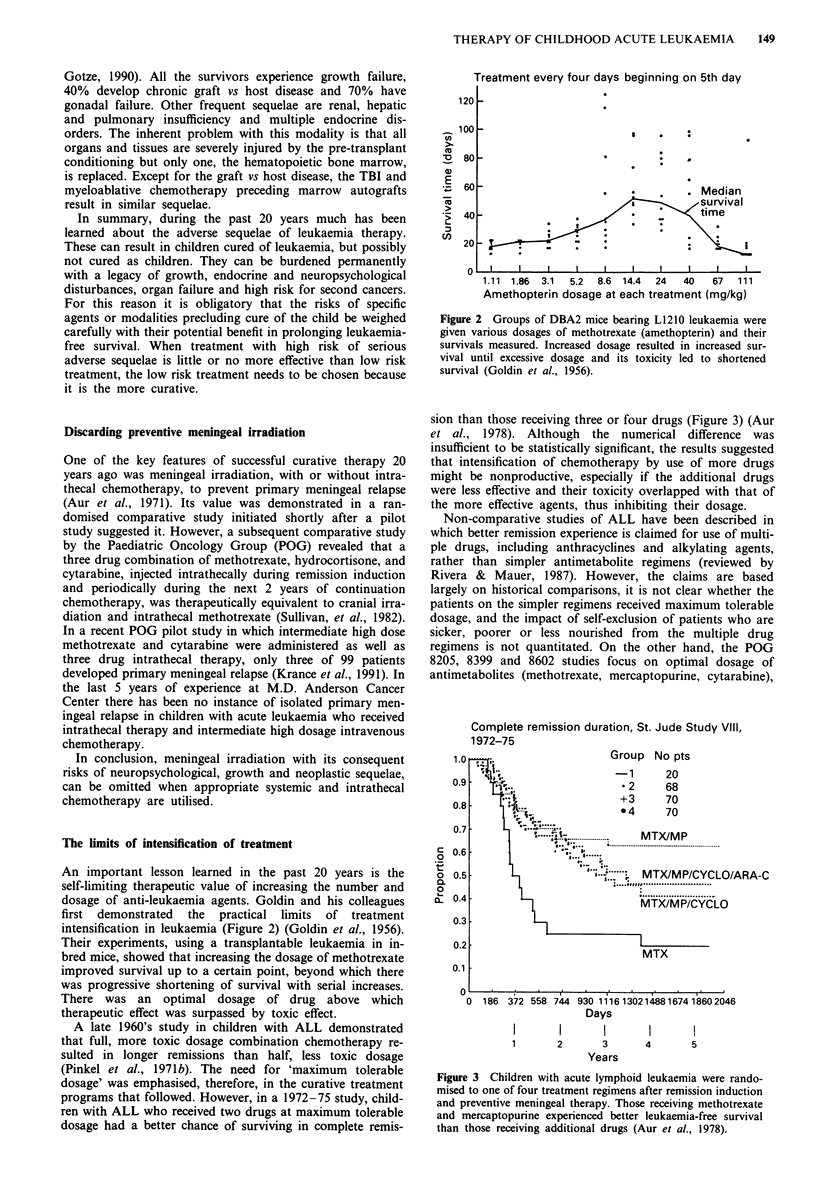

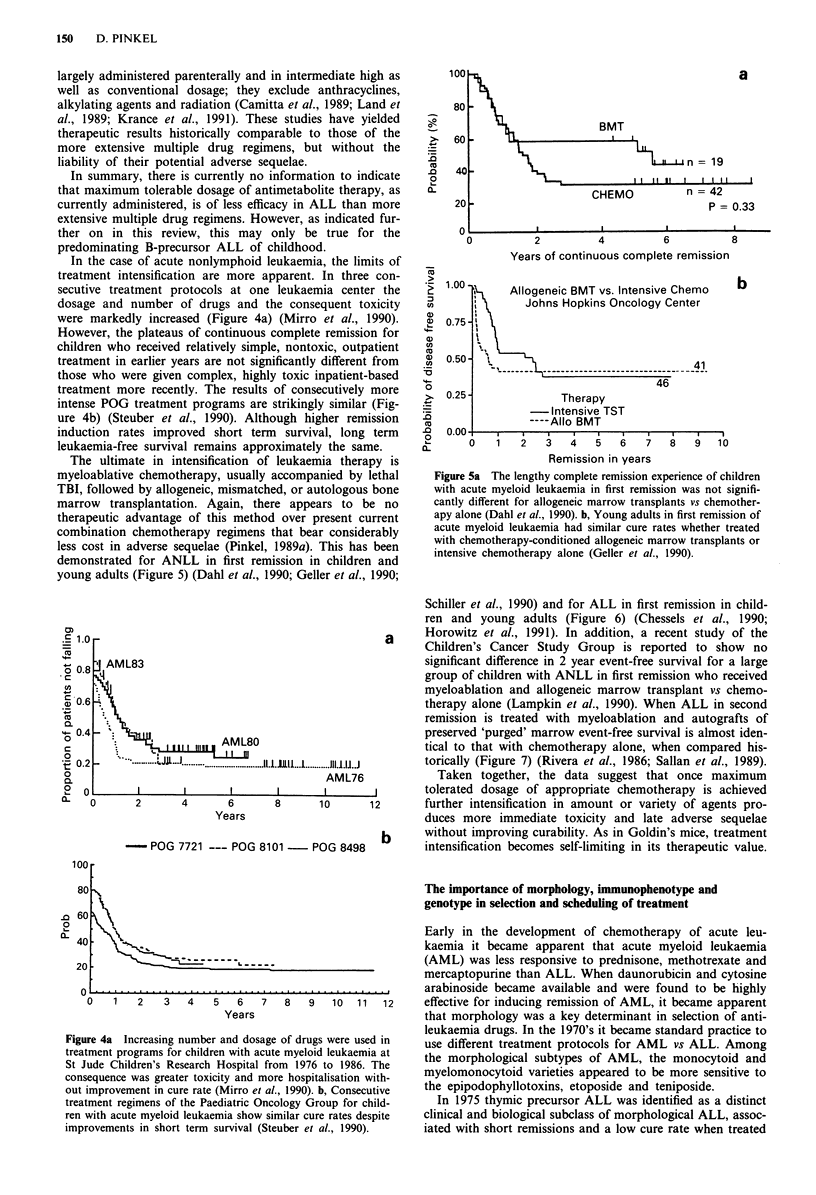

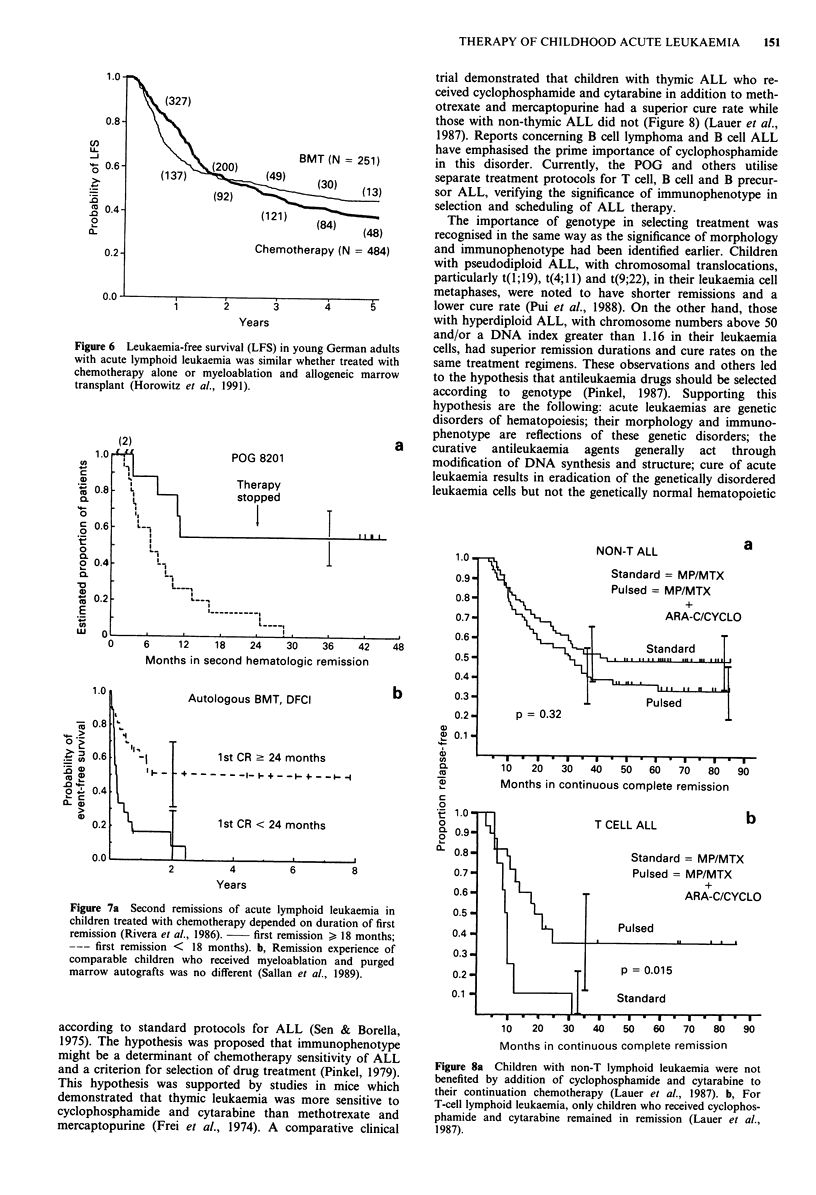

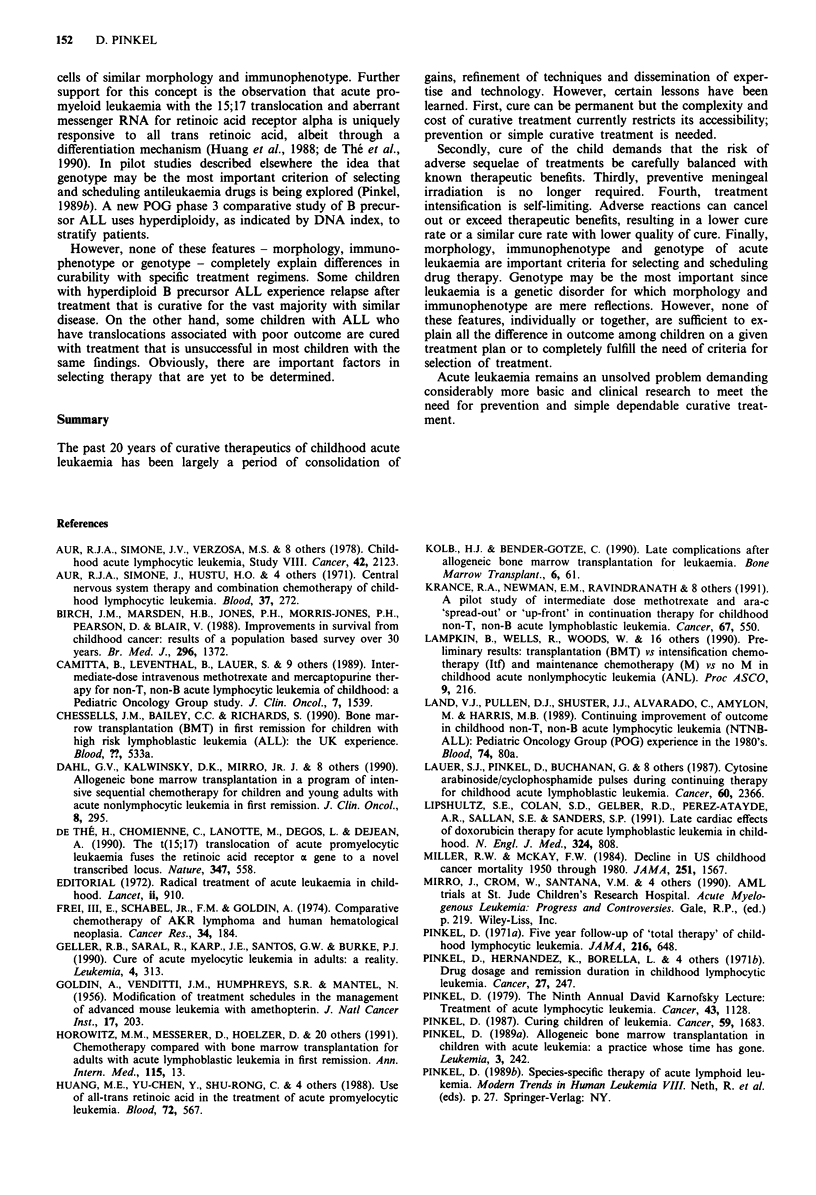

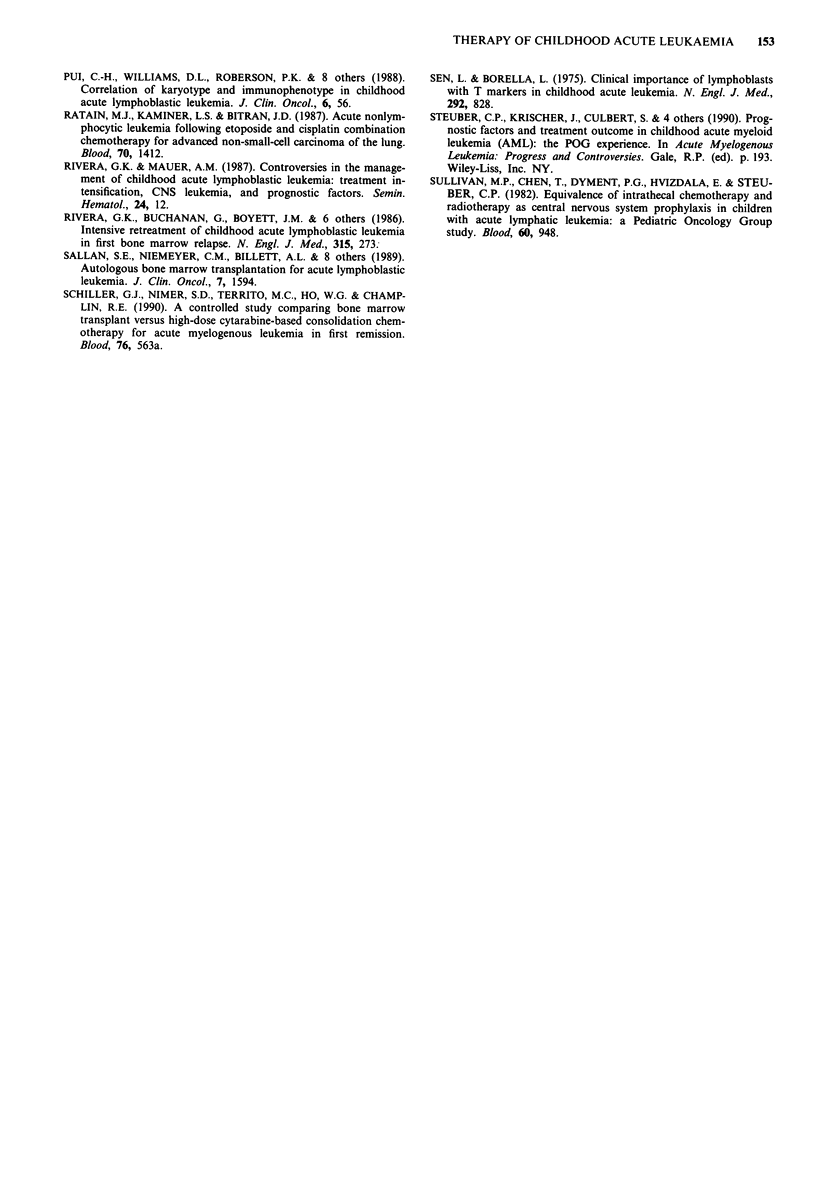


## References

[OCR_00790] Aur R. J., Simone J. V., Verzosa M. S., Hustu H. O., Barker L. F., Pinkel D. P., Rivera G., Dahl G. V., Wood A., Stagner S. (1978). Childhood acute lymphocytic leukemia: study VIII.. Cancer.

[OCR_00793] Aur R. J., Simone J., Hustu H. O., Walters T., Borella L., Pratt C., Pinkel D. (1971). Central nervous system therapy and combination chemotherapy of childhood lymphocytic leukemia.. Blood.

[OCR_00798] Birch J. M., Marsden H. B., Jones P. H., Pearson D., Blair V. (1988). Improvements in survival from childhood cancer: results of a population based survey over 30 years.. Br Med J (Clin Res Ed).

[OCR_00806] Camitta B., Leventhal B., Lauer S., Shuster J. J., Adair S., Casper J., Civin C., Graham M., Mahoney D., Munoz L. (1989). Intermediate-dose intravenous methotrexate and mercaptopurine therapy for non-T, non-B acute lymphocytic leukemia of childhood: a Pediatric Oncology Group study.. J Clin Oncol.

[OCR_00816] Dahl G. V., Kalwinsky D. K., Mirro J., Look A. T., Pui C. H., Murphy S. B., Mason C., Ruggiero M., Schell M., Johnson F. L. (1990). Allogeneic bone marrow transplantation in a program of intensive sequential chemotherapy for children and young adults with acute nonlymphocytic leukemia in first remission.. J Clin Oncol.

[OCR_00833] Frei E., Schabel F. M., Goldin A. (1974). Comparative chemotherapy of AKR lymphoma and human hematological neoplasia.. Cancer Res.

[OCR_00843] GOLDIN A., HUMPHREYS S. R., MANTEL N., VENDITTI J. M. (1956). Modification of treatment of schedules in the management of advanced mouse leukemia with amethopterin.. J Natl Cancer Inst.

[OCR_00838] Geller R. B., Saral R., Karp J. E., Santos G. W., Burke P. J. (1990). Cure of acute myelocytic leukemia in adults: a reality.. Leukemia.

[OCR_00855] Huang M. E., Ye Y. C., Chen S. R., Chai J. R., Lu J. X., Zhoa L., Gu L. J., Wang Z. Y. (1988). Use of all-trans retinoic acid in the treatment of acute promyelocytic leukemia.. Blood.

[OCR_00867] Krance R. A., Newman E. M., Ravindranath Y., Harris M. B., Brecher M., Wimmer R., Shuster J. J., Land V. J., Pullen J., Crist W. (1991). A pilot study of intermediate-dose methotrexate and cytosine arabinoside, "spread-out" or "up-front," in continuation therapy for childhood non-T, non-B acute lymphoblastic leukemia. A Pediatric Oncology Group study.. Cancer.

[OCR_00885] Lauer S. J., Pinkel D., Buchanan G. R., Sartain P., Cornet J. M., Krance R., Borella L. D., Casper J. T., Kun L. E., Hoffman R. G. (1987). Cytosine arabinoside/cyclophosphamide pulses during continuation therapy for childhood acute lymphoblastic leukemia. Potential selective effect in T-cell leukemia.. Cancer.

[OCR_00890] Lipshultz S. E., Colan S. D., Gelber R. D., Perez-Atayde A. R., Sallan S. E., Sanders S. P. (1991). Late cardiac effects of doxorubicin therapy for acute lymphoblastic leukemia in childhood.. N Engl J Med.

[OCR_00896] Miller R. W., McKay F. W. (1984). Decline in US childhood cancer mortality. 1950 through 1980.. JAMA.

[OCR_00920] Pinkel D. (1989). Allogeneic bone marrow transplantation in children with acute leukemia: a practice whose time has gone.. Leukemia.

[OCR_00919] Pinkel D. (1987). Curing children of leukemia.. Cancer.

[OCR_00906] Pinkel D. (1971). Five-year follow-up of "total therapy" of childhood lymphocytic leukemia.. JAMA.

[OCR_00910] Pinkel D., Hernandez K., Borella L., Holton C., Aur R., Samoy G., Pratt C. (1971). Drug dosage and remission duration in childhood lymphocytic leukemia.. Cancer.

[OCR_00915] Pinkel D. (1979). The ninth annual David Karnofsky Lecture. Treatment of acute lymphocytic leukemia.. Cancer.

[OCR_00937] Ratain M. J., Kaminer L. S., Bitran J. D., Larson R. A., Le Beau M. M., Skosey C., Purl S., Hoffman P. C., Wade J., Vardiman J. W. (1987). Acute nonlymphocytic leukemia following etoposide and cisplatin combination chemotherapy for advanced non-small-cell carcinoma of the lung.. Blood.

[OCR_00951] Rivera G. K., Buchanan G., Boyett J. M., Camitta B., Ochs J., Kalwinsky D., Amylon M., Vietti T. J., Crist W. M. (1986). Intensive retreatment of childhood acute lymphoblastic leukemia in first bone marrow relapse. A Pediatric Oncology Group Study.. N Engl J Med.

[OCR_00943] Rivera G. K., Mauer A. M. (1987). Controversies in the management of childhood acute lymphoblastic leukemia: treatment intensification, CNS leukemia, and prognostic factors.. Semin Hematol.

[OCR_00954] Sallan S. E., Niemeyer C. M., Billett A. L., Lipton J. M., Tarbell N. J., Gelber R. D., Murray C., Pittinger T. P., Wolfe L. C., Bast R. C. (1989). Autologous bone marrow transplantation for acute lymphoblastic leukemia.. J Clin Oncol.

[OCR_00966] Sen L., Borella L. (1975). Clinical importance of lymphoblasts with T markers in childhood acute leukemia.. N Engl J Med.

[OCR_00980] Sullivan M. P., Chen T., Dyment P. G., Hvizdala E., Steuber C. P. (1982). Equivalence of intrathecal chemotherapy and radiotherapy as central nervous system prophylaxis in children with acute lymphatic leukemia: a pediatric oncology group study.. Blood.

[OCR_00823] de Thé H., Chomienne C., Lanotte M., Degos L., Dejean A. (1990). The t(15;17) translocation of acute promyelocytic leukaemia fuses the retinoic acid receptor alpha gene to a novel transcribed locus.. Nature.

